# Numerical characterization of intraoperative and chronic electrodes in deep brain stimulation

**DOI:** 10.3389/fncom.2015.00002

**Published:** 2015-02-19

**Authors:** Alessandra Paffi, Francesca Camera, Francesca Apollonio, Guglielmo d’Inzeo, Micaela Liberti

**Affiliations:** Department of Information Engineering, Electronics and Telecommunications (DIET), Sapienza University of RomeRome, Italy

**Keywords:** deep brain stimulation, intraoperative electrode, chronic electrode, numerical model, stimulation efficacy

## Abstract

An intraoperative electrode (microelectrode) is used in the deep brain stimulation (DBS) technique to pinpoint the brain target and to choose the best parameters for the electrical stimulus. However, when the intraoperative electrode is replaced with the chronic one (macroelectrode), the observed effects do not always coincide with predictions. To investigate the causes of such discrepancies, a 3D model of the basal ganglia has been considered and realistic models of both intraoperative and chronic electrodes have been developed and numerically solved. Results of simulations of the electric potential (V) and the activating function (AF) along neuronal fibers show that the different geometries and sizes of the two electrodes do not change the distributions and polarities of these functions, but rather the amplitudes. This effect is similar to the one produced by the presence of different tissue layers (edema or glial tissue) in the peri-electrode space. Conversely, an inaccurate positioning of the chronic electrode with respect to the intraoperative one (electric centers not coincident) may induce a completely different electric stimulation in some groups of fibers.

## Introduction

Deep brain stimulation (DBS) is an effective symptomatic treatment for several movement disorders, such as Parkinson’s disease (PD), essential tremor (ET), and dystonia (Okun et al., 2012). It is an invasive stimulation technique, where a biphasic pulsed electric stimulus is delivered by implanted electrodes to the basal ganglia, the brain region associated with the control of voluntary motor movements. In particular, the brain nuclei mostly chosen as the stimulation targets are the Subthalamic Nucleus (STN) for the PD, and the Globus Pallidus (Gp) and Ventral Intermediate (VIM) nucleus for the ET (Limousin and Martinez-Torres, 2008; Benabid et al., 2009; Chopra et al., 2013; Lozano and Hallett, 2013; Okun and Zeilman, 2014). Ongoing translational research seeks to identify safe and effective new brain targets and new stimulation paradigms for the treatment of movement and affective disorders (Kringelbach et al., 2007). The DBS stimulus, whose main parameters are the amplitude, the pulse width, and the repetition frequency, is generated by a neurostimulator also called implanted pulse generator (IPG) surgically implanted near the collarbone (Okun and Zeilman, 2014).

Despite its effectiveness, similarly to other techniques involving electric or magnetic stimulation (Di Lazzaro et al., 2013), the interaction mechanisms of the DBS signal with neuronal circuits are not clearly understood (McIntyre et al., 2004a; Deniau et al., 2010; Shah and Schiff, 2010), and the neural response to the stimulation is not yet fully predictable. In general, to have a reliable estimate of the electric or magnetic stimulation of the nervous system, the first unavoidable step is the dosimetric computation of the electrical potential inside the brain tissue (Apollonio et al., 2000, 2013; Joucla et al., 2014). The dosimetric computation can be performed by numerically solving the electromagnetic problem inside realistic brain models, stimulated by simplified or realistic electrodes (McIntyre and Grill, 2002; Butson and McIntyre, 2006; Bossetti et al., 2008; Grant and Lowery, 2010; Joucla et al., 2012a,b; Wongsarnpigoon and Grill, 2012; Paffi et al., 2013b). Then the extracellular electrical stimulation must be coupled with neuronal models (Giannì et al., 2005; Miocinovic et al., 2006; Paffi et al., 2013a) to obtain the actual neuronal response (Joucla et al., 2014).

In clinical practice, the correct brain region to be stimulated and the optimal parameters for the stimulation signal are empirically chosen using an intraoperative microelectrode in both recording and stimulation configurations. The recording phase is used to pinpoint the target site, whereas stimulation allows the neurosurgeon to determine the best parameters for the DBS signal to improve the symptoms while controlling side effects (Okun and Zeilman, 2014). After the microelectrode recording locates the precise target, the neurosurgeon puts the chronic electrode in place and connects the lead to the IPG, suitably programmed using the chosen electric parameters. However, after replacing the intraoperative electrode with the chronic one, the observed effects are often very different from predictions (Lafreniere-Roula et al., 2009), and a long phase of parameters tuning, carried out by reprogramming the IPG, is generally needed.

In previous studies (Maggio et al., 2010; Paffi et al., 2013b,c), the authors used a realistic numerical model of the basal ganglia (Maggio et al., 2009) and a simplified model of the electrodes to conclude that the observed discrepancy between intraoperative and chronic stimulation could be due to the relative positioning between micro- and macroelectrodes. In particular, the electric potential (V) and the activating function (AF; Rattay, 1998) calculated along neuronal fibers revealed that the chronic and the intraoperative electrodes produced the same effects only if their electric centers coincided, otherwise, the fibers response could be completely different. In that work, both chronic and intraoperative electrodes were modeled with two active contacts with octahedral shape. The only difference between the two kinds of electrodes was in their size: the active surface of the chronic electrode was three orders of magnitude larger than that of the intraoperative one. However, the actual geometry of commonly used intraoperative electrodes (FHC) is much more complicated, including different active contacts to be used in recording or stimulating configurations (FHC, 2013; Lempka and McIntyre, 2013). In a recent study (Lempka and McIntyre, 2013) a 2D realistic model of the FHC 5005 Z electrode was developed to characterize its recording properties.

Moreover, recent studies showed that the properties of the tissue layer near the electrode surface are essential to simulate electric stimulation in a realistic way (Joucla and Yvert, 2009; Lempka and McIntyre, 2013) since they may change the contact impedance and thus the stimulation efficacy. This layer is significantly different, in terms of electric properties and thickness, for the chronic and intraoperative electrodes and may significantly change during the first weeks of implantation (Kent and Grill, 2014; Okun and Zeilman, 2014).

For these reasons, here we have developed realistic models of the 3389 Medtronic chronic electrode and the 5005 Z FHC intraoperative electrode, accounting for the actual geometries and materials, using the commercial software Comsol Multiphysics. The chronic and intraoperative electrodes have been placed in different relative positions and the layer of edema or of encapsulating tissue at the interface between electrodes and brain tissue has been included.

The aim of this work is to compare simulations of intraoperative and chronic electrodes in terms of impedance, V and AF, in order to identify the influence of geometry, positioning, and electric properties of the interface layer. These dosimetric results will help in interpreting the discrepancies observed in clinics between intraoperative and chronic stimulations. Thus, in perspective, such information could usefully support the neurosurgeon in the correct placement of the permanent electrode and in the fast remodulation of parameters to be used in the chronic stimulation.

## Materials and methods

### Modeling of the volume conductor

**Figure 1:**
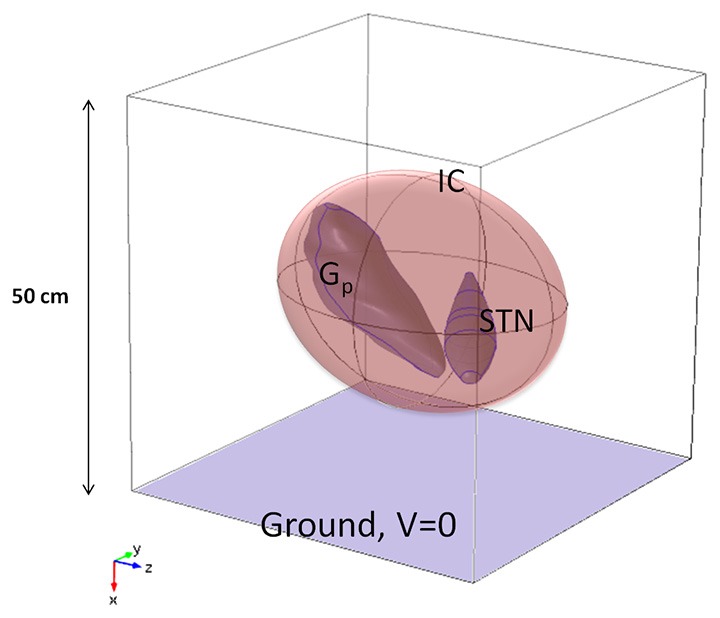
**Model of the neuroanatomic target, including STN, Gp, and IC, inside a cubic box 50 cm of side (not in scale)**.

The 3D model (Maggio et al., 2009; Paffi et al., 2013b) of the target was obtained from clinical MRI data of the basal ganglia (Maggio et al., 2009) and encompasses the STN, the Gp, and the internal capsule (IC). The main dimension of the STN is 17.4 mm, that of the Gp 23.7 mm, and the IC is a spheroidal region of white matter with semi-major axis equal to 19 mm, surrounding the STN and the Gp. These anatomical regions are particularly important in DBS since neural activity between the STN and the Gp (Lozano and Mahant, 2004; McIntyre et al., 2004a) is impaired in PD, and the IC contains bundles of long fibers connecting the STN and the Gp. The geometric model of these nuclei was imported into Comsol Multiphysics v.4.4 (Comsol Inc), based on Finite Element Methods (FEM), and inserted at the center of a cubic box (Figure 1), 50 cm on a side (Maggio et al., 2009; Paffi et al., 2013b).

Following Kuncel and Grill (2004), McIntyre et al. (2004b), and Maggio et al. (2009), the Gp and the STN were modeled as isotropic gray matter (*σ* = 0.2 S/m), whereas anisotropic properties were assigned to the IC (*σ*_yy_ = *σ*_xx_ = 0.1 S/m, *σ*_zz_ = 1 S/m) (Kuncel and Grill, 2004; Wakana et al., 2004; Liberti et al., 2007) with the maximum value of conductivity in the fiber direction, i.e., almost parallel to the z-axes of the Cartesian reference system (Figure 1). Following Maggio et al. (2009), the box domain was filled with an isotropic medium representative of the bulk brain tissue at a frequency of around 100 Hz (*σ* = 0.09 S/m) (Gabriel et al., 1996).

Due to the quasi-static nature of the problem (McIntyre et al., 2004a; Miocinovic et al., 2006; Joucla et al., 2014), the Laplace equation was solved using the AC/DC Electric Currents module.

### Modeling of the intraoperative electrode

The intraoperative electrode, often referred to as the microelectrode, has the twofold function of pinpointing the target site, if used in recording configuration, and identifying the best signal parameters for the stimulation. In this study we considered the commercial electrode FHC 5005 Z (FHC, 2013). It has a geometry consisting of coaxial cylinders with different radii and lengths. The inner cylinder of platinum (radius of 62.5 µm) looks like a needle that protrudes from the outer covering structure with a length ranging from 1 to 10 mm. It terminates at the distal extremity in a cone, 388 µm long, having a tip angle of about 18°. The surface of 1250 µm^2^ of area at the tip of the cone is used as an active contact (cathode) during the recording phase and is usually referred to as “micro” (FHC, 2013; Lempka and McIntyre, 2013). Except for the protruding part, the needle is covered by an insulating material (epoxy resin: *σ* = 1 × 10^−16^ S/m) with outer radius of 200 µm. Two shields of steel, outer radius of 280 µm, cover the insulating material both at the distal and proximal ends; the distal metal clamp, 1 mm long, is often referred to as “macro” and is separated from the remaining shield (“cannula”) by the same insulation material, 1 mm tall.

**Figure 2:**
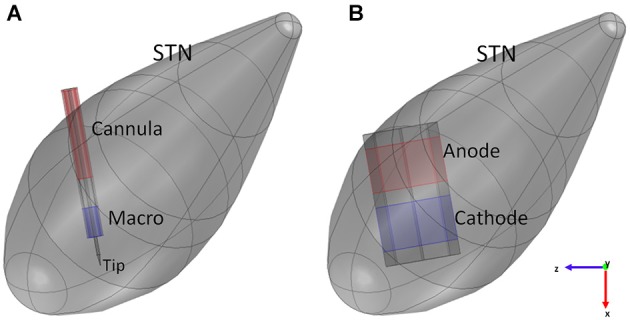
**Models of the intraoperative electrode (A) and the chronic one (B) inside the STN**. Blue surfaces represent the cathode, red surfaces the anode of the electrodes.

During recordings, “macro” represents the other active contact (anode), conversely, in the stimulating configuration here considered (Figure 2A), the two active contacts are represented by “macro” (cathode) and “cannula” (anode) (FHC, 2013).

According to the previous description, the intraoperative electrode was modeled in Comsol 4.4 (Figure 2A), fixing the protrusion of the needle at the minimum value (1 mm); moreover, the proximal part of the lead was truncated with a cap of steel, so that the overall length of the modeled electrode was 6.05 mm. The assigned materials were platinum (*σ* = 8.6 × 10^6^ S/m) for the needle, epoxy resin (*σ* = 1 × 10^−16^ S/m) for the insulating material, and steel (*σ* = 4.032 × 10^6^ S/m) for “macro” and “cannula”.

Since in this study the attention is focused on the stimulation, the chosen active contacts were “macro” and “cannula” in the bipolar configuration, so that –1 V was assigned to the “macro” (cathode), +1 V to the “cannula” (anode) and the ground to one face of the analysis box.

The position of the electric center between the active contacts of the intraoperative electrode was calculated by finding the location along the electrode axis where the V became null. Due to the asymmetry between the anode and cathode, the electric center does not fall in the exact middle between the two.

### Modeling of the chronic electrode

The modeled chronic electrode was the Medtronic “3389” one (Medtronic manual), having the external diameter of 1.27 mm, and active contacts 1.5 mm tall, with inter-distance of 0.5 mm. Two adjacent active contacts were taken into account, and modeled as platinum solids (*σ* = 8.6 × 10^6^ S/m) with octagonal section (Sel et al., 2003). With respect to a circular section, this shape permits a simpler discretization of the surfaces, thus minimizing numerical errors (Sel et al., 2003). The same octahedral shape was adopted for the insulating material (*σ* = 1 × 10^−12^ S/m) placed between the active contacts and at the other two extremities of the contacts (Figure 2B); each insulating domain was 0.5 mm long.

In this work bipolar stimulation was considered, that is the two active contacts were set to a positive (anode) and a negative (cathode) voltage, respectively, while the ground condition was applied to one face of the cubic box (Maggio et al., 2009). This configuration is the same used for the intraoperative electrode and provides a narrower and more focused stimulation, with the maximum effect near the cathode (Volkmann et al., 2002).

Since therapeutic amplitudes for DBS signals normally range between 1 and 4 V, the cathode was set to −1 V, and the anode to +1 V (Figure 2), according to the specifications reported in Shipton (2012) and Medtronic manual.

**Figure 3:**
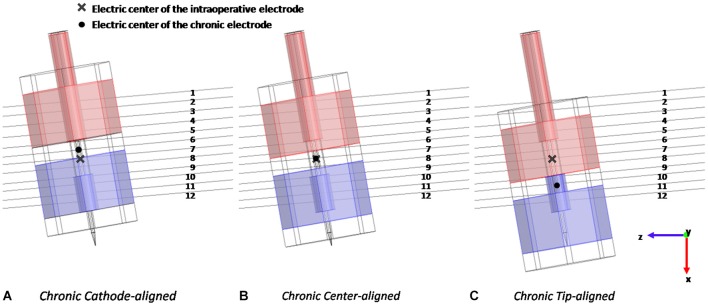
**Different positions of the chronic electrode with respect to the intraoperative one and to the 12 considered fibers: with the cathodes tangent to each other (A), with the electric centers coincident (B), and with the cathode of the chronic electrode tangent to the tip of the intraoperative one (C)**.

The stimulation was studied with the chronic electrode placed in three different positions with respect to the intraoperative electrode: *Cathode-aligned, Center-aligned*, and *Tip-aligned*, as shown in Figure 3. In one placement (Figure 3B) the electric center of the chronic electrode is coincident with the electric center of the intraoperative one (*Center-aligned*). The other two placements are with the cathode of the chronic electrode tangent to the tip of the microelectrode (*Tip-aligned*) (Figure 3C) and with the cathodes of the macro and microelectrode tangent to each other (*Cathode-aligned*) (Figure 3A).

In the *Tip-aligned* position the electric center of the chronic electrode is shifted from that of the intraoperative one of 0.692 mm, whereas, in the case of the *Cathode-aligned*, the electric center is shifted of 0.300 mm in the opposite direction (Figure 3).

According to the clinical practice for PD, in all considered positions the active contacts are inside the STN; only in the *Cathode-aligned* configuration a part of the anode protrudes towards the IC.

The EM solutions were obtained using a mesh with more than 1 × 10^6^ tetrahedral elements. Due to the great difference in dimensions of different sub-domains, the mesh density was set in a nonuniform way. The minimum element size is set to 1 µm, and the maximum to 17.5 mm; the maximum growth rate between an element and an adjacent one is set to 1.35, for a total number of 53016 tetrahedrons for the intraoperative electrode model and 8735 for the chronic one.

### Modeling of the electrode-tissue layer

The insertion of the electrodes in the brain inevitably induces the formation of a thin layer immediately adjacent to the electrode surface with electric properties often very different from those of the gray matter of the STN. In particular, during the intraoperative phase it is likely to find layers of edema made of blood and/or serum; on the contrary, during the chronic stimulation, a glial encapsulation tissue grows in the peri-electrode space (Kent and Grill, 2014).

Experimental (Lempka et al., 2009) and theoretical studies (Joucla et al., 2014) have shown that the properties of this layer strongly affect the electrode impedance, which, in turns, changes the current/voltage relation.

Some IPGs keep the delivered voltage constant and the injected current varies; postoperative increase in tissue impedance imposes a voltage increase to have the same electric current injected in the tissue.

According to (Lempka and McIntyre, 2013; Kent and Grill, 2014), these layers were modeled as a 100 µm (Lempka and McIntyre, 2013) or 500 µm (Kent and Grill, 2014) peri-electrode space to represent the edema (*σ* = 1.7 S/m) around the intraoperative electrode or the glial encapsulation layer (*σ* = 0.1 S/m) around the chronic one (Butson et al., 2006; Yousif et al., 2008).

### Observables

Results of the simulations conducted with the two types of electrode are based on the evaluation and comparison of the distribution of V and AF along the 12 lines of Figure 3, representative of the fibers direction, passing through the Gp and the STN. The AF is defined as the second derivative of the extracellular potential along a fiber (Rattay, 1998). On the basis of classical cable theory it has been argued that long and straight nervous fibers may be activated in the regions where the AF assumes a positive value (Rattay, 1998). Conversely, where AF is negative, possible fiber inhibition occurs. The threshold value for the AF, able to induce activation or inhibition of the fiber, depends on the specific features of the fiber and may be defined only coupling the dosimetric analysis with neuronal modeling (Warman et al., 1992). However, in the absence of such a threshold, the sign of the AF is useful to qualitatively estimate the regions of depolarization and hyperpolarization generated by the stimulating electrode on neuronal fibers (Rattay, 1998).

Another important property characterizing the electrode functioning is the impedance. In particular, in the bipolar configuration here considered, three different impedances can be calculated: the differential impedance between the active contacts, and the common mode impedance between each contact and ground. These values were calculated by applying the superposition principle and by setting the terminal boundary condition to the outer faces of the active contacts and ground. This procedure allowed us to separate the common mode from the differential electric currents.

## Results: chronic vs. intraoperative stimulation

### The effect of geometry

The first simulations were carried out with the intraoperative and chronic electrodes placed with the electric centers coincident (Figure 3B). In this way it was possible to study the effect of the two different geometries and sizes on the observed electrical quantities.

The impedance calculation (Table 1) reveals that the two common mode impedances (R_cath_ and R_an_) are almost equal for the chronic electrode, reflecting the perfect symmetry of the geometries of the two active contacts. On the contrary, for the intraoperative electrode, R_cath_ is three times R_an_ according to the different stimulating areas of “macro” and “cannula”. The differential impedances (R_diff_) of the two electrodes allow us to make a first comparison between the intraoperative and the chronic bipolar stimulation. Table 1 shows that R_diff_ of the intraoperative electrode is about six times that of the chronic one, according to the different sizes of the contact surfaces. This allows us to predict that with the same potentials applied to the contacts the voltage induced in the target region will be lower in the intraoperative stimulation than in the chronic one.

**Table 1:** **Impedance values between the two active contacts (R_diff_), between cathode and ground (R_cath_), and between anode and ground (R_an_) for the intraoperative and the chronic electrodes**.

	Intraoperative electrode	Chronic electrode
R_diff_ (kΩ)	2.53	0.44
R_cath_ (kΩ)	5.08	2.54
R_an_ (kΩ)	1.82	2.37

**Figure 4:**
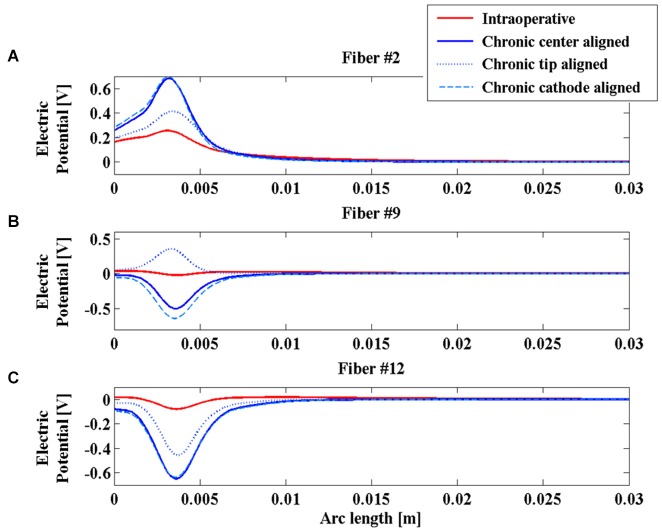
**Electric potential (V) along fibers #2 (A), #9 (B), and #12 (C) for the intraoperative electrode (solid red line), the chronic electrode in the *Center-aligned* position (blue solid line), the chronic electrode in the *Tip-aligned* position (blue dotted line), and the chronic electrode in the *Cathode-aligned* position (cyan dashed line)**.

Looking at the V induced along the lines, Figure 4 shows significant examples of three fibers in different positions with respect to the active contacts. Fibers #2, #9 and #12 were selected (Figure 3A).

Comparing the two red and blue solid lines of Figure 4, one can see that the polarity and the whole distributions are very similar for the two electrodes along the considered fibers, moreover it is possible to confirm the predicted difference in the V amplitude between the chronic and the intraoperative electrodes, and a slight widening of the bell shaped curve relative to this last one.

**Figure 5:**
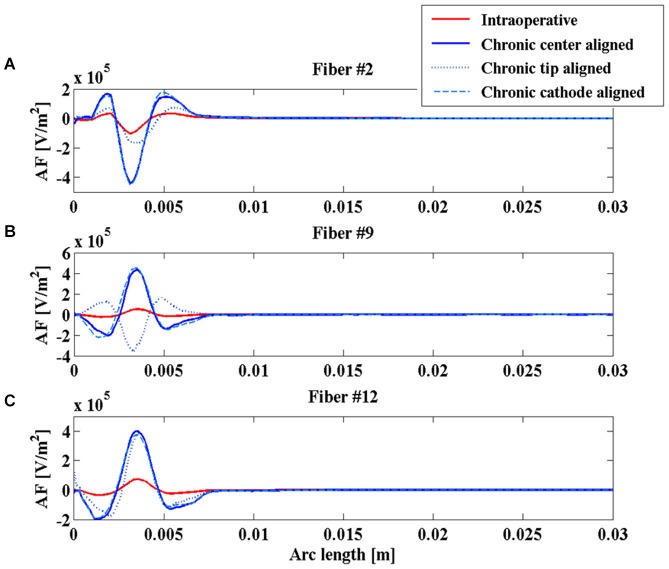
**Activating function (AF) along fibers #2 (A), #9 (B), and #12 (C) for the intraoperative electrode (solid red line), the chronic electrode in the *Center-aligned* position (blue solid line), the chronic electrode in the *Tip-aligned* position (blue dotted line), and the chronic electrode in the *Cathode-aligned* position (cyan dashed line)**.

Even the AFs (red and blue solid lines of Figure 5) show similar behaviors along the considered fibers, suggesting that the different geometries of the two electrodes do not significantly affect their stimulation properties once that the two electric centers are aligned.

### The effect of positioning

In Paffi et al. (2013b) the authors identified in an imprecise positioning of the chronic electrode with respect to the intraoperative electrode an important source of discrepancies between the effects of the two stimulations. In that work, simplified models of the intraoperative and chronic electrodes had the same geometries but very different sizes. In the present paper the authors want to evaluate whether this effect of positioning holds even in the more realistic case where the two electrodes have different geometries but more similar dimensions of the active contacts. Indeed, even in this case, the particular shape of the intraoperative electrode, with the needle protruding from the cannula, gives rise to possible inaccuracies in the chronic electrode placement, as shown in Figures 3A,C.

As shown in Figure 4A, the V along the fiber #2 always assumes positive values, since the fiber passes close to the anode of both intraoperative and chronic electrodes, independently of the positioning of the latter (Figure 3). Similarly, the AFs in all considered stimulation conditions show triphasic behaviors with the same signs, corresponding to the same portions of the fiber (Figure 5A). Similar behaviors are shown for fibers #1–#6 (data not shown).

Conversely, for fibers #7–#10 the electrode positioning becomes critical, since the fibers of this group may lie at one or the other side of the electric center, depending on the electrode position. As an example, we show the potentials V along the fiber #9 (Figure 4B), which are all negative except for the chronic electrode in the *Tip-aligned* configuration. Indeed, only in this case, fiber #9 passes closer to the cathode. As a consequence, the AF shows an opposite behavior with respect to the other configurations, with positive peaks where the other configurations showed negative peaks and vice versa (Figure 5B).

For fibers #11 and #12 (see Figure 4C) all the Vs assume negative values and the AFs show similar trends (Figure 5C).

These data confirm our previous results regarding the importance of a correct positioning of the chronic electrode (Paffi et al., 2013b). If the electric centers of the intraoperative and chronic electrodes do not coincide, there is a group of fibers that may exhibit a response to the chronic stimulation that is completely different from that observed during the intraoperative stimulation.

### The effect of the layer between electrode and tissue

To further compare intraoperative and chronic stimulations, realistic conditions for the layer at the interface electrode-tissue were considered, as described in Section Modeling of the electrode-tissue layer. In particular, the intraoperative electrode was realistically simulated surrounded by an edema layer of two different thicknesses (100 and 500 µm), whereas the chronic electrode was simulated in the *Center-aligned* position inside a layer of glial encapsulating tissue 100 or 500 µm thick.

In Table 2 the differential impedances of both electrodes are reported in all the considered conditions. The obtained values clearly show that the presence of the edema in the peri-electrode space decreases the electrode impedance, due to the higher value of the conductivity with respect to that of the STN (1.7 S/m vs. 0.2 S/m). This effect increases with the layer thickness. In a similar way, the presence of the glial encapsulating tissue (*σ* = 0.1 S/m) around the chronic electrode increases its impedance, degrading the stimulation efficacy as the layer thickness increases.

**Table 2:** **Differential impedance (R_diff_) of the intraoperative electrode surrounded by 100 or 500 µm of edema and of the chronic electrode surrounded by 100 or 500 µm of glial tissue**.

	Intraoperative electrode		Chronic electrode	
	Edema 100 µm	Edema 500 µm	Glia 100 µm	Glia 500 µm
R_diff_ (kΩ)	0.90	0.27	0.66	0.94

**Figure 6:**
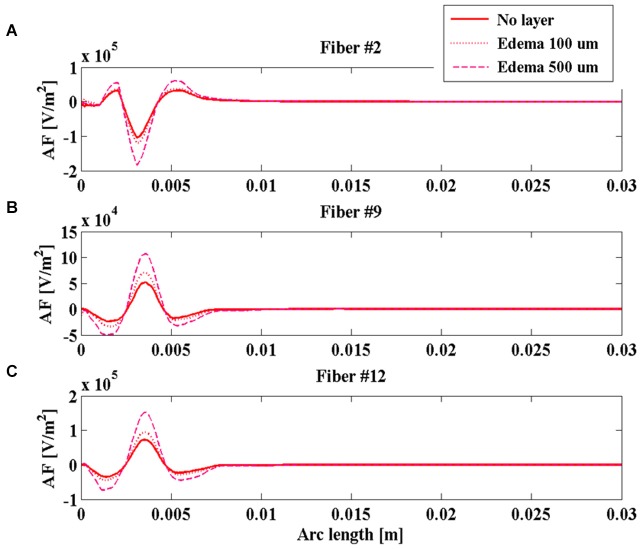
**Activating function (AF) along fibers #2 (A), #9 (B), and #12 (C) for the intraoperative electrode in the absence of edema layer (red solid line) and in the presence of an edema layer 100 µm (red dotted line) or 500 µm thick (magenta dashed line)**.

During the intraoperative stimulation, the effect of the edema on the stimulation is shown in Figure 6 for fibers #2 (Figure 6A), #9 (Figure 6B), and #12 (Figure 6C). According to the calculated impedances, the edema makes the peaks of the AF become much more pronounced, especially for the layer 500 µm thick. In this case, the absolute value of the main peak increases more than 80% for all considered fibers, making the intraoperative stimulation more similar to the chronic one.

**Figure 7:**
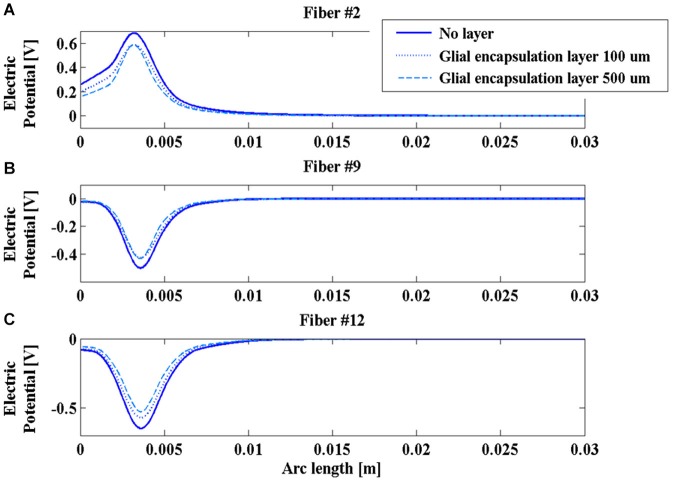
**Electric potential (V) along fibers #2 (A), #9 (B), and #12 (C) for the chronic electrode in the absence of glial layer (blue solid line) and in the presence of a glial layer 100 µm (blue dotted line) or 500 µm thick (cyan dashed line)**.

On the contrary, the presence of the glial layer around the chronic electrode induces a decrease of the V along the fibers (Figure 7) that is much more sensitive to the change in conductivity than to the layer thickness.

This is a possible cause of the decrease in time of the efficacy of the DBS stimulation, so that new techniques, such as the closed loop DBS, have been developing to adaptively change the stimulation parameters during the chronic therapy (Winestone et al., 2012; Kent and Grill, 2014).

## Discussion and conclusions

In this work, realistic 3D numeric models of both intraoperative (5005 Z FHC) and chronic (3389 Medtronic) DBS electrodes have been developed and their stimulation features have been studied, taking into account possible inaccuracies in the positioning of the chronic electrode and the presence of edema or glial layers at the electrode-tissue interface. The electrode performances in the different studied conditions have been compared in terms of impedance and suitable electric quantities (V and AF) inside the brain nuclei along lines representative of neuronal fibers connecting the STN and the Gp, in order to identify which parameters mainly affect the stimulation characteristics (e.g., magnitude, AF sign etc.) and consequently can be used to interpret the clinically observed discrepancies between intraoperative and chronic stimulation.

The obtained results indicate that the impedance of the intraoperative electrode is higher than that of the chronic one (Table 1). Therefore, if the same voltage difference is applied between the active contacts, both V and AF assume higher values in chronic stimulation. The effect of the edema layer surrounding the intraoperative electrode is a decrease of the impedance (Table 2) with a better efficacy of stimulation; both V and AF increase in amplitude and approach the values obtained with chronic stimulation (Figure 6). Therefore, we conclude that the edema layer can help in obtaining similar results between chronic and intraoperative stimulation. Conversely, the glial encapsulating layer induces an opposite effect on the chronic stimulation, with increasing impedance (Table 2) and decreasing values of V (Figure 7). The formation of this layer during the first weeks of implantation could be one of the causes of a progressive degradation of the DBS performance that often requires the IPG reprogramming. To overcome this limit, particularly interesting is the closed-loop DBS where the signal parameters are adaptively adjusted on the bases of electrophysiological traces recorded using the same chronic electrode (Winestone et al., 2012; Kent and Grill, 2014).

Great emphasis has been placed on the accurate modeling of the geometries of the two electrodes that greatly differ both in terms of dimensions and shapes, since this was a plausible cause of the different clinical results. Simulations clearly show that the two electrodes give similar results in terms of shapes and polarities of V and AF if their electric centers coincide (Figures 4, 5). Conversely, the displacement of the chronic electrode with respect to the electric center of the intraoperative electrode may lead to the inversion of polarity of V and AF on a specific set of fibers (Figures 4, 5). This seems the most interesting result in order to understand the actual distributions on the brain nuclei of the electric stimulations due to the two electrodes. These results confirm the importance, already shown by the author using simplified electrode models (Paffi et al., 2013b,c), of an accurate positioning of the chronic electrode when replacing the intraoperative one, in order to avoid different stimulation results.

Finally we conclude that the presence of an electrode interface layer can explain discrepancies between intraoperative and chronic stimulation only for the case of the glial one after some weeks following the implantation. More important, in particular reference to the AF sign, we can interpret our results to mean that to assure that all fibers receive the same kind of stimulation, the chronic electrode must be placed accurately, taking note of the exact position of the electric center of the intraoperative electrode.

## Author contributions

All the authors contributed to the paper design, data analysis and paper drafting.

## Conflict of interest statement

The authors declare that the research was conducted in the absence of any commercial or financial relationships that could be construed as a potential conflict of interest.
